# Mitochondrial involvement in PC: improving therapeutic strategies

**DOI:** 10.3389/fphar.2025.1710923

**Published:** 2025-11-07

**Authors:** Jiaxin Zhang, Chang Lou

**Affiliations:** Department of Intensive Care Unit, Beilun People’s Hospital, Ningbo, Zhejiang, China

**Keywords:** prostate cancer, oxidative phosphorylation, mitochondria DNA, reactiveoxygen species, membrane permeabilization, apoptosis, mitochondrial dynamics

## Abstract

Prostate cancer (PC) is a complex disease propelled by various molecular mechanisms. The role of mitochondria in PC has recently emerged as a significant research focus. Mitochondria, often referred to as the cell’s powerhouses, are not only essential for energy production but also crucial for key cellular processes like apoptosis, oxidative stress, and metabolic reprogramming. Changes in energy metabolism, marked by an increased dependency on oxidative phosphorylation (OXPHOS), have been noted in PC cells, offering a potential therapeutic target. Moreover, specific mitochondrial DNA (mtDNA) mutations have been linked with advanced tumors and adverse patient outcomes in PC. The mitochondrial reactive oxygen species (ROS), the disruption of mitochondrial dynamics and the fine balance between pro-apoptotic and anti-apoptotic signals mediated by Bcl-2 family proteins have also been implicated in PC. Comprehending the complex interaction between mitochondria and PC biology offers substantial potential for creating innovative targeted therapeutic strategies. This review emphasizes the role of mitochondria in the occurrence and malignant progression of PC, as well as the potential of targeted interventions on mitochondria in developing treatments, which may improve the prognosis of PC patients.

## Graphical Abstract

**Figure:**
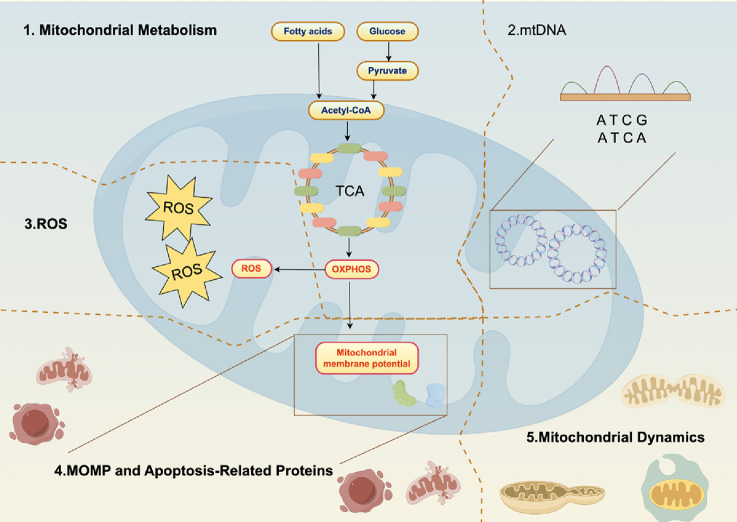

## 1 Introduction

Prostate cancer (PC) is a complex disease propelled by diverse molecular mechanisms that contribute to its onset and progression (Wasim et al., 2022). The role of mitochondria in PC has recently emerged as a significant research focus (Zhu et al., 2017). Mitochondria, often referred to as the powerhouses of the cell, are not only essential for energy production but also functions as a master regulator of key cellular processes such as apoptosis, oxidative stress, and metabolic reprogramming in PC (Mukherjee et al., 2023).

A critical aspect of mitochondrial involvement in PC is the regulation of energy metabolism (Burke, 2017). Altered metabolic phenotypes, marked by an increased dependency on oxidative phosphorylation (OXPHOS) for adenosine triphosphate (ATP) production over glycolysis, have been noted in PC cells (Massie et al., 2011). This metabolic shift presents a promising therapeutic target, as selectively disrupting mitochondrial function and OXPHOS may effectively inhibit PC cell growth and survival (Mahmoud et al., 2023; Chen et al., 2021b). Mitochondrial DNA (mtDNA) has also been implicated in PC (Borah et al., 2023). Specific mtDNA mutations have been linked with advanced tumors and adverse patient outcomes in PC (Chouchani et al., 2016; Kalsbeek et al., 2016; Hopkins et al., 2017). These mtDNA mutations can interact with events driven by the nuclear genome, thereby contributing to the development and progression of PC (Sharma and Sampath, 2019). Oxidative stress, primarily mediated by reactive oxygen species (ROS) generated in mitochondria, plays a dual role in PC (Joshi et al., 2020; Lee et al., 2023). ROS can promote tumor progression by activating signaling pathways involved in cell proliferation and survival, thereby playing a role in the advancement of PC (Joshi et al., 2020). However, excessive levels of ROS can induce cellular damage and trigger apoptosis (Lee et al., 2023). Targeting the regulation of mitochondrial ROS represents a potential approach to restore redox balance and selectively induce apoptosis in PC cells (Perez-Gomez et al., 2023). Apoptosis, a tightly regulated programmed cell death process, is intricately controlled by mitochondria (Desagher and Martinou, 2000). Mitochondrial outer membrane permeabilization (MOMP) and the subsequent release of apoptogenic factors, including cytochrome c, play crucial roles in initiating apoptosis (Karch et al., 2015). Dysregulation of MOMP and alterations in the expression of Bcl-2 family proteins, which regulate the delicate balance between pro-apoptotic and anti-apoptotic signals, have been observed in PC (Doroshenko et al., 2022). A comprehensive understanding of the mechanisms underlying mitochondrial-mediated apoptosis resistance holds promise for the development of targeted therapies aimed at overcoming chemoresistance and improving treatment outcomes in PC. Furthermore, maintaining mitochondrial function and cellular homeostasis relies on crucial processes of mitochondrial dynamics, including fusion, fission, and mitophagy (Chan, 2020). Targeting mitochondrial dynamics has emerged as a viable therapeutic strategy that may offer a novel approach to induce apoptosis and inhibit tumor growth in PC (Deng et al., 2022; Park et al., 2020).

In conclusion, mitochondria play a multifaceted role in PC, influencing various aspects such as energy metabolism, mtDNA regulation, oxidative stress, apoptosis, and mitochondrial dynamics. Understanding the complex interplay between mitochondria and PC biology holds great promise for the development of innovative diagnostic tools and targeted therapeutic approaches. Continued exploration of the potential of mitochondria as therapeutic targets may pave the way for more effective treatments for PC.

## 2 Overview of OXPHOS in PC

### 2.1 Role of OXPHOS in PC metabolism

Unlike most cancers that exhibit the Warburg effect (aerobic glycolysis), PC displays a unique metabolic profile (Vander Heiden et al., 2009). In normal prostate epithelium, high zinc levels inhibit mitochondrial aconitase, truncating the tricarboxylic acid (TCA) cycle and suppressing OXPHOS, leading to citrate secretion (Chen et al., 2021b; Fontana et al., 2023; Massie et al., 2011). During carcinogenesis, diminished zinc levels restore aconitase activity, enabling resumed citrate oxidation, TCA cycle flux, and heightened OXPHOS dependence for ATP production in early-stage PC (Fontana et al., 2023; Vayalil and Landar, 2015; Wanjari et al., 2023). This OXPHOS reliance is further supported by clinical evidence: PC tissues show increased mitochondrial mass, and OXPHOS complex expression correlates with Gleason score (Feichtinger et al., 2018). Notably, circulating tumor cells (CTCs) from PC patients exhibit elevated OXPHOS, facilitating survival and dissemination (Jiang et al., 2024).

Even in advanced, hormone-refractory PC (HRPC), where the Warburg effect is often observed, OXPHOS remains active and contributes to progression (Fontana et al., 2023). For instance, cannabidiol and cannabigerol can increase glycolytic capacity and inhibit OXPHOS in HRPC cells, demonstrating strong anti-tumor effects in in vivo models (Mahmoud et al., 2023). This suggests that the importance of targeting OXPHOS in PC. Besides, AR-active subtypes of castration-resistant PC (ARPC) are highly dependent on OXPHOS, while more aggressive variant PCs (AVPC) tend to rely on glycolysis (Mossa et al., 2023). This metabolic heterogeneity is closely linked to the tumor microenvironment: in bone metastasis sites, PC cells engage in a “reverse Warburg effect” through interactions with stromal cells. In this process, cancer-associated fibroblasts (CAFs) secrete metabolites such as lactate, which fuel OXPHOS in tumor cells (Xiong et al., 2025). Therefore, investigating the regulatory mechanisms of mitochondrial energy metabolism in PC remains highly significant. A deeper understanding of these metabolic pathways may help unravel the processes of cancer initiation and progression, offering new therapeutic opportunities by targeting mechanisms that contribute to the development and maintenance of malignant phenotypes in PC.

### 2.2 Regulatory mechanisms of OXPHOS in PC

#### 2.2.1 Transcriptional regulation

Multiple transcription factors and coactivators play crucial roles in regulating OXPHOS in PC. Peroxisome proliferator-activated receptor gamma coactivator 1-alpha (PGC-1α), a key regulator of mitochondrial biogenesis, is upregulated in small cell neuroendocrine PC (SCNC) (Varuzhanyan et al., 2024). Studies demonstrate that PGC-1α forms a positive feedback loop with the neuroendocrine differentiation marker ASCL1, enhancing mitochondrial biogenesis and OXPHOS activity, thereby driving SCNC progression and defining the ASCL1 subtype (Varuzhanyan et al., 2024). TEAD4, a core transcription factor in the Hippo pathway, has recently been identified to regulate OXPHOS independently of YAP1. Evidence indicates that arginine modulates histone acetylation via epigenetic mechanisms, facilitating the recruitment of TEAD4 to promoter/enhancer regions of OXPHOS genes and promoting the upregulation of nuclear-encoded OXPHOS components (Chen et al., 2021a). Silencing TEAD4 suppresses OXPHOS function and inhibits PC cell growth both *in vitro* and *in vivo*. Estrogen-related receptor alpha (ERRα) is a key regulator of energy metabolism regulation in PC stem cells (PCSCs). ERRα enhances the stemness and metabolic reprogramming of PCSCs by trans-repressing the zinc transporter ZIP1—reducing intracellular zinc uptake—and trans-activating ACO2 (m-aconitase), thereby restoring the TCA cycle (Ma et al., 2025).

#### 2.2.2 Metabolic regulation

The metabolic state of PC cells is finely tuned by various metabolic enzymes and transporters. The mitochondrial pyruvate carrier (MPC), responsible for transporting pyruvate from the cytosol into the mitochondrial matrix, is a critical determinant of metabolic flux. Genetic knockout of MPC1 or pharmacological inhibition with UK5099 attenuates OXPHOS, enhances glycolysis, and promotes stem-like properties and chemoresistance in PC cells (Li et al., 2017; Zhong et al., 2015). The pyruvate dehydrogenase complex (PDHc), which bridges glycolysis and the TCA cycle, also plays a pivotal role. Reduced expression of PDHA1 (the E1α subunit of PDHc) is associated with increased stemness and poor prognosis (Zhong et al., 2017). PDHA1 knockout cells exhibit diminished mitochondrial OXPHOS and enhanced glycolysis, accompanied by chemoresistance, increased migratory capacity, and elevated expression of cancer stem cell markers. The succinate dehydrogenase (SDH) complex, particularly its subunit B (SDHB), contributes significantly to metabolic adaptation in PC. Following resistance to androgen receptor signaling inhibitors (ARSI), elevated levels of SDHB mRNA have been detected in serum extracellular vesicles (EVs), suggesting a potential role in the development of drug resistance (Kato et al., 2024).

#### 2.2.3 Genetic regulation

Mutations in mtDNA contribute to OXPHOS remodeling in PC. High-grade prostate tumors harbor an elevated burden of potentially deleterious mtDNA mutations, which correlate with adverse risk factors (Schopf et al., 2020). A high load of such mutations in complex I-encoding genes is associated with a 70% reduction in NADH pathway capacity and a compensatory increase in succinate pathway activity. Alterations in the expression of nuclear-encoded OXPHOS subunits also influence disease progression. Reduced expression of the ATP synthase subunit ATP5F1A has been linked to early-onset PC (Feichtinger et al., 2018). Isolated or combined deficiencies in OXPHOS complexes are observed in approximately 17% of PC and 18% of benign prostate tissues. Complex I deficiency is present in 9% of samples, and ATP5F1A is the most frequently affected subunit, altered in 10% of tumors and 11% of benign prostate tissues (Feichtinger et al., 2018).

### 2.3 Diagnostic and prognostic biomarkers

Proteins and genes associated with OXPHOS hold significant prognostic value. Studies have found that elevated expression of NDUFS1 and ATP5O is significantly associated with earlier biochemical recurrence (Wiebringhaus et al., 2021). The mRNA levels of these genes are substantially higher in prostatic intraepithelial neoplasia and PC tissues compared to benign prostatic glands. Pyruvate dehydrogenase kinase 4 (PDK4) expression serves as an independent prognostic marker, predicting disease recurrence regardless of established diagnostic risk factors such as Gleason grade, clinical stage, and PSA levels (Oberhuber et al., 2020). Low PDK4 expression represents a promising biomarker for identifying aggressive PC with poor outcomes.

The levels of OXPHOS-related mRNAs in EVs correlate with their expression levels in PC tissues. EV-derived SDHB shows potential for the early detection of resistance to androgen receptor signaling inhibitors (ARSI) (Kato et al., 2024). In a study of seven patients treated with ARSI, three whose PSA levels initially stabilized later developed resistant castration-resistant PC (CRPC), and these patients exhibited increased EV-SDHB. Furthermore, the mitochondrial oncobiogenic index (MOBI), a mathematically derived representation of tumor bioenergetic profile, increases significantly during prostate tumorigenesis but declines rapidly below normal levels as cells become invasive (Vayalil and Landar, 2015). MOBI appears to be associated with cancer stage rather than androgen dependency or mitochondrial content, suggesting its potential use as a biomarker to distinguish between aggressive and indolent disease.

### 2.4 Targeting strategies

**FIGURE 1:**
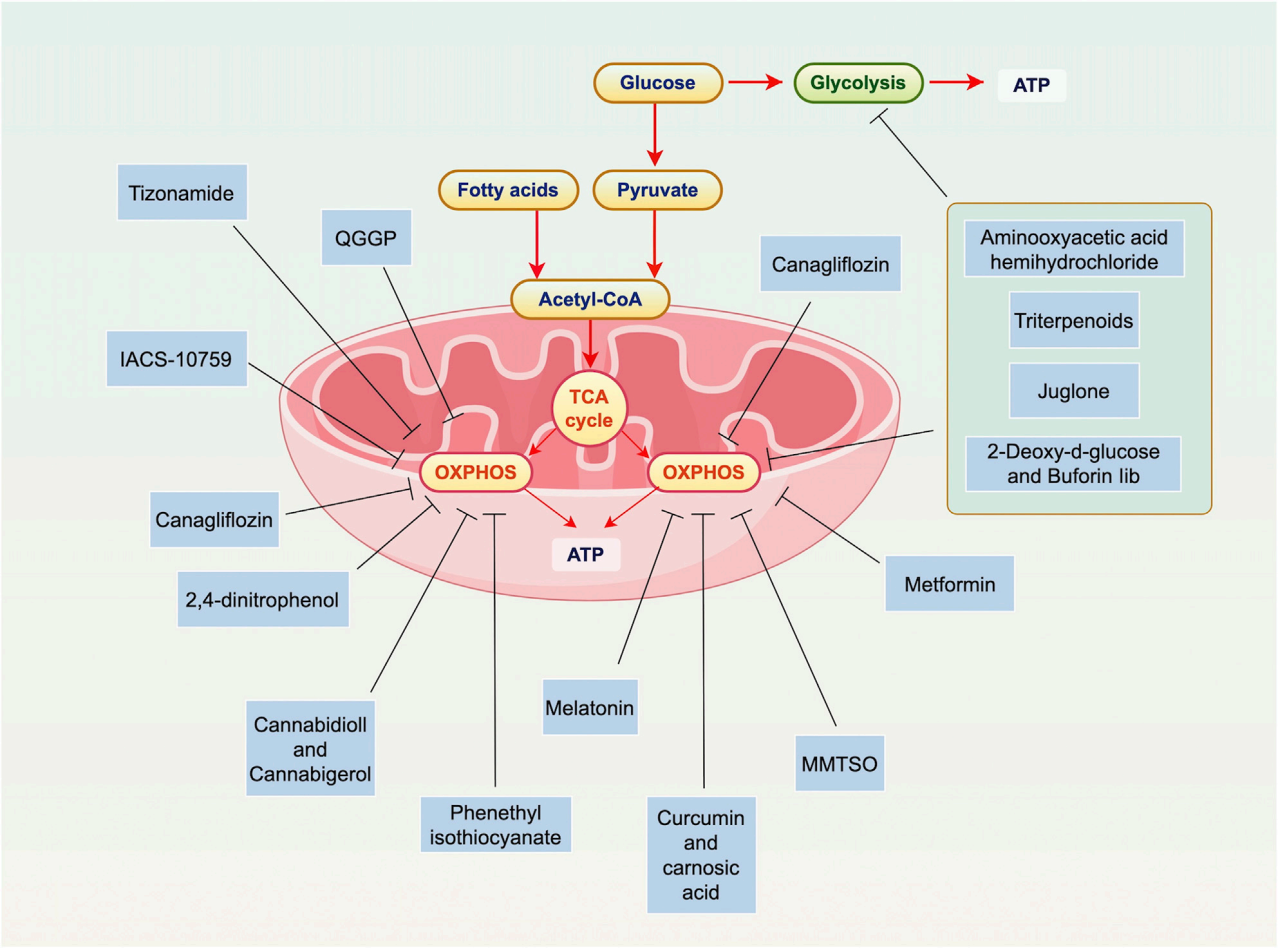
Overview of OXPHOS in PC. Mitochondria are key to PC cell metabolism, especially in the shift from glycolysis to OXPHOS and lipogenesis. In this process, glucose is converted to pyruvate, which is then oxidized in the mitochondria to form acetyl-CoA. This enters the TCA cycle, producing ATP through OXPHOS. Various therapies target mitochondrial metabolism in PC, including inhibiting ATP production, blocking the unfolded protein response, and disrupting OXPHOS. Specific interventions like Mannose, Canagliflozin, and 2,4-dinitrophenol have shown promise. Dual targeting of both mitochondrial metabolism and glycolysis could help overcome cancer cell resistance, such as Triterpenoids (Oleanolic Acid, Nummularic Acid, and Plectranthoic Acid), Aminooxyacetic acid hemihydrochloride, 2-Deoxy-d-glucose, buforin Iib. OXPHOS is particularly important in certain types of PC, like HRPC. Preliminary evidence supports the use of OXPHOS-targeting drugs, such as metformin. These findings highlight the potential of targeting mitochondrial metabolism in PC treatment. Abbreviations: ATP: adenosine triphosphate; TCA: tricarboxylic acid cycle; OXPHOS: oxidative phosphorylation; ATP: adenosine triphosphate; TCA: tricarboxylic acid cycle; MMTSO: metabolite S-methyl methanethiosulfonate; IQGAP1: IQ motif-containing GTPase-activating protein 1; QGGP: Quercetin 3-O-(6″-galactopyranosyl)-β-D-galactopyranoside.

Due to the critical role of OXPHOS in metabolism and cell survival, targeting OXPHOS in PC is an attractive therapeutic strategy (Figure 1; Table 1).

**TABLE 1:** Targeting OXPHOS in PC.

Targeted drug or treatment	Primary mechanism	Current limitations/Challenges	Translational readiness	Ref.
Cannabidiol and cannabigerol	Inhibit ETC complexes, increase ROS, deplete ATP	Low bioavailability, relatively non-specific targeting	Primarily preclinical research	Mahmoud et al. (2023)
Mannose				Deng et al. (2022)
Phenethyl isothiocyanate				Xiao et al. (2010)
Curcumin and carnosic acid				Ossikbayeva et al. (2021)
Melatonin	Inhibit ETC complexes, suppress TCA cycle/OXPHOS, reduce mitochondrial ATP production	Insufficient pharmacokinetic data	Preclinical research	Chen et al. (2025)
MMTSO		challenges in achieving effective anticancer doses		Beasy et al. (2025)
Juglone	Simultaneously inhibit OXPHOS and glycolysis	Potential systemic energy toxicity, undefined therapeutic window	Preclinical research	Hu et al. (2023)
Triterpenoids (Oleanolic Acid, Nummularic Acid, Plectranthoic Acid)				Liu et al. (2014), Younis et al. (2018), Woods et al. (2003)
Aminooxyacetic acid hemihydrochloride				Teng et al. (2023)
2-Deoxy-d-glucose and Buforin Iib				Jang et al. (2015)
QGGP	Disrupts CAF-PC cell crosstalk, inhibits mitochondrial biogenesis	May show limited efficacy against PC with tumor-autonomous OXPHOS dependency	Preclinical research	Xiong et al. (2025)
IACS-10759	Inhibit ETC Complex I	Variable efficacy against metabolically heterogeneous tumors	IACS-10759 (Clinical stage), Metformin (Under clinical investigation)	Mossa et al. (2023)
Metformin				Ippolito et al. (2016)
Canagliflozin	Blocks mitochondrial OXPHOS function	Primary indication for diabetes, anticancer efficacy being evaluated	Drug repurposing phase	Ali et al. (2023)
2,4-dinitrophenol	Disrupt proton gradient, deplete ATP	Narrow therapeutic window, historical safety concerns	Preclinical research	Adamczuk et al. (2022)
Tizonamide				Hawsawi et al. (2025)

Abbreviations: OXPHOS: oxidative phosphorylation; HRPC: hormone-refractory prostate cancer; PC: prostate cancer; ATP: adenosine triphosphate; TCA: tricarboxylic acid cycle; MMTSO: metabolite S-methyl methanethiosulfonate; QGGP: Quercetin 3-O-(6″-galactopyranosyl)-β-D-galactopyranoside.

#### 2.4.1 Natural compounds

A variety of natural compounds exhibit anti-PC activity by modulating OXPHOS. Plant-derived cannabinoids have been used as palliative care agents for cancer patients for decades (Blaskovich et al., 2021). Cannabidiol and cannabigerol can increase glycolytic capacity and inhibit OXPHOS in HRPC cells, demonstrating strong anti-tumor effects in in vivo models (Mahmoud et al., 2023). Mannose, an isomer of glucose, can inhibit the progression of PC by reducing the production of mitochondrial ATP (Deng et al., 2022). Phenethyl isothiocyanate induces PC cell death by inhibiting complex III activity and OXPHOS, leading to increased ROS production and ATP depletion (Xiao et al., 2010). Curcumin and carnosic acid, at low concentrations, synergistically inhibit the proliferation of metastatic PC cells and alter mitochondrial function (Ossikbayeva et al., 2021). Additionally, mitochondria-targeted melatonin potently suppresses the TCA cycle and OXPHOS, thereby enhancing ROS generation, disrupting mitochondrial membrane potential, and ultimately inducing tumor cell pyropoptosis (Chen et al., 2025). S-Methyl-L-cysteine sulfoxide (SMCSO) and its metabolite S-methyl methanethiosulfonate (MMTSO) are sulfur-containing compounds found in cruciferous and allium vegetables. Studies have shown that MMTSO reduces mitochondrial metabolism, mitochondrial ATP production, and the proportion of OXPHOS in DU145 PC cells, while simultaneously increasing cellular dependence on fatty acids (Beasy et al., 2025). Transcriptomic and metabolomic analyses further reveal that MMTSO reprograms energy metabolism pathways and modulates immune responses, shifting cancer cells toward a less aggressive phenotype. These findings provide a mechanistic basis for dietary interventions in PC prevention and management (Beasy et al., 2025).

The metabolic flexibility of cancer cells may upregulate compensatory pathways, such as glycolysis, to support cancer cell survival when mitochondrial metabolism is inhibited (Pavlova and Thompson, 2016). Therefore, compounds that can simultaneously target mitochondrial metabolism and glycolysis may help overcome this resistance mechanism. Juglone, a natural compound, suppresses both OXPHOS and glycolysis by inhibiting the activity of key glycolytic enzymes including hexokinase (HK), phosphofructokinase (PFK), and pyruvate kinase (PK) (Hu et al., 2023). Triterpenoids are metabolic products of isopentenyl pyrophosphate (IPP) oligomers, with known members exceeding 20,000 (Liu et al., 2014). Oleanolic Acid, Nummularic Acid, and Plectranthoic Acid can inhibit both glycolysis and mitochondrial OXPHOS, induce apoptosis in PC cells, and inhibit cell proliferation activity (Liu et al., 2014; Younis et al., 2018; Woods et al., 2003). Aminooxyacetic acid hemihydrochloride can target both glycolysis and mitochondrial OXPHOS, inhibit ATP production, and induce apoptosis in PC cells (Teng et al., 2023). In addition, combined treatment with 2-Deoxy-d-glucose and buforin Iib can effectively induce apoptosis in PC cells, with minimal cytotoxicity to normal cells (Wanyan et al., 2020). 2-Deoxy-d-glucose is a commonly used glycolysis inhibitor (Zhang et al., 2014). Buforin IIb has a mitochondrial targeting effect (Jang et al., 2015). Dual targeting of glycolysis and mitochondria may be an effective anticancer strategy for treating PC.

Non-malignant cells within the TME, such as CAFs play critical roles in PC progression and therapy resistance (Fan et al., 2025). Specifically, angiopoietin-like protein 4 secreted by CAFs binds to IQ motif-containing GTPase-activating protein 1 (IQGAP1) on PC cells, activating the Raf-MEK-ERK-PGC1α signaling axis. This promotes mitochondrial biogenesis and OXPHOS metabolism, thereby enhancing cell proliferation and conferring resistance to chemotherapy (Xiong et al., 2025). The natural flavonoid glycoside Quercetin 3-O-(6″-galactopyranosyl)-β-D-galactopyranoside (QGGP) has been identified as an inhibitor of IQGAP1, promoting its degradation. Combination treatment with QGGP and docetaxel (DTX) reverses CAF-mediated chemoresistance and improves therapeutic efficacy (Xiong et al., 2025).

#### 2.4.2 Drugs

Targeting OXPHOS has emerged as a promising therapeutic strategy in PC. IACS-10759, a clinical-grade OXPHOS inhibitor, demonstrates significant efficacy in suppressing OXPHOS-dependent ARPC cell lines (Mossa et al., 2023). *In vivo* studies further reveal that IACS-10759 inhibits the growth of both subcutaneous and bone-localized C4-2B tumors. However, it exhibits no effect on subcutaneous PC3 tumors, underscoring the impact of metabolic heterogeneity and the tumor microenvironment on treatment response (Mossa et al., 2023). Canagliflozin is an inhibitor of sodium-glucose cotransporter-2, approved for the treatment of diabetes and heart failure (Neal et al., 2017). Canagliflozin can block mitochondrial OXPHOS and inhibit the survival of androgen-sensitive and insensitive human PC cells (Ali et al., 2023). In addition, in some preclinical models, combining drugs targeting mitochondrial metabolism with chemotherapy has achieved good therapeutic effects. One strategy for treating advanced cancer is to target the energy metabolism of cancer cells (Kroemer and Pouyssegur, 2008). The compound 2,4-dinitrophenol disrupts cellular OXPHOS by interfering with its ability to metabolize energy (Geisler, 2019). 2,4-dinitrophenol has a significant synergistic effect when combined with the chemotherapy drugs doxorubicin and epirubicin (Adamczuk et al., 2022). Mechanistically, this may be related to inhibiting ATP synthesis in PC cells and simultaneously producing excessive ROS to damage mitochondria (Adamczuk et al., 2022). Mitochondrial uncouplers such as tizonamide inhibit OXPHOS by dissipating the proton gradient across the inner mitochondrial membrane. This disruption depletes cellular ATP levels, leading to activation of the AMPK-p38 signaling pathway. Subsequent downstream events include degradation of cyclin D1, dephosphorylation of Rb, and ultimately suppression of the transcription factor E2F1 activity (Hawsawi et al., 2025). As a critical oncoprotein, E2F1 regulates the expression of genes involved in cell cycle progression, DNA synthesis, and lipid metabolism. Its inhibition significantly impedes the proliferation of PC cells (Hawsawi et al., 2025). Additionally, OXPHOS complex I inhibitors such as metformin have also demonstrated anti-tumor potential in PC (Pujalte-Martin et al., 2024). In preclinical models, PC cells resistant to docetaxel exhibit a metabolic shift from glycolysis to OXPHOS, and metformin can inhibit Complex I of OXPHOS in PC, suppressing the progression of PC (Ippolito et al., 2016).

Finally, the effects of targeting OXPHOS drugs have been preliminarily confirmed in clinical studies (Chen et al., 2021b). Regarding clinical studies, the anti-cancer effects of metformin have been extensively evaluated in various types of PC, including patients with or without combined radiotherapy, chemotherapy, or androgen deprivation therapy (ADT) (Trials: NCT01243385, NCT01620593, NCT01796028, NCT01677897). In castration-resistant patients treated with metformin alone, over 50% of patients showed optimistic PSA responses (Trial: NCT01243385). Overall, targeting pathways related to mitochondrial metabolism will contribute to the development of mitochondrial inhibitors for inhibiting PC, ushering in a new era of cancer treatment.

### 2.5 Opportunities and challenges in targeting OXPHOS

Despite the considerable promise of targeting OXPHOS in PC treatment, emerging evidence indicates that its therapeutic efficacy is strongly context-dependent. This dependency manifests primarily in two key areas: disease stage/molecular subtype and the tumor microenvironment.

First, the disease stage and molecular subtype significantly influence OXPHOS dependence. Early-stage PC cells develop OXPHOS reliance through zinc-mediated metabolic reprogramming, while advanced disease demonstrates substantial heterogeneity (Chen et al., 2021b; Fontana et al., 2023; Massie et al., 2011). Specifically, the AR-active CRPC subtype maintains strong OXPHOS dependence, whereas more AVPC preferentially utilize glycolysis (Mossa et al., 2023). This metabolic heterogeneity directly translates to differential treatment responses, as exemplified by IACS-10759 showing efficacy in OXPHOS-dependent models but not in glycolysis-reliant ones (Mossa et al., 2023). These findings underscore the critical need for metabolic subtyping before initiating OXPHOS-targeted therapies.

Second, the tumor microenvironment plays a crucial modulatory role. Through the “reverse Warburg effect,” cancer cells can leverage metabolites secreted by CAFs—such as lactate—to sustain their own OXPHOS metabolism (Xiong et al., 2025). This mechanism explains why strategies solely targeting tumor-intrinsic mitochondrial function may fail and emphasizes the therapeutic potential of simultaneously disrupting stromal-tumor metabolic crosstalk.

Moreover, metabolic plasticity represents another major therapeutic challenge (Pavlova and Thompson, 2016). Inhibition of OXPHOS frequently triggers compensatory glycolytic enhancement, as demonstrated following MPC suppression (Li et al., 2017; Zhong et al., 2015), leading to treatment resistance. Therefore, the most promising future approaches likely involve combining OXPHOS inhibitors with either glycolysis inhibitors or standard chemotherapeutic agents.

Significant hurdles remain, particularly the current absence of reliable biomarkers for identifying OXPHOS-dependent patient subgroups. The development of robust subtyping tools—potentially based on metabolic imaging, functional analysis of circulating tumor cells, or serum metabolomic profiling—constitutes an essential prerequisite for successful clinical translation. Additionally, for natural compounds targeting OXPHOS, improving their bioavailability and target specificity remains a priority for future research.

## 3 Overview of mtDNA in PC

### 3.1 Characteristics of mtDNA in PC

Cancer development and progression involve the accumulation of genomic sequence alterations (Zong et al., 2016). Human mtDNA is a circular double-stranded DNA consisting of 16,500 nucleotides, including 37 coding genes that encode subunits of mitochondrial electron transport chain complexes, 22 transfer RNAs, and two ribosomal RNAs (Kopinski et al., 2021). Most mtDNA is homoplasmic at birth, meaning there is high consistency between copies (Filograna et al., 2021). However, the mitochondrial genome is devoid of protective histones and robust DNA repair mechanisms, rendering it highly susceptible to DNA damage (Kauppila et al., 2017). Due to frequent exposure to high levels of ROS and a lack of sufficient proofreading and repair mechanisms, the mutation rate of mtDNA is approximately 10–100 times higher than that of nuclear genome DNA (Smith et al., 2022). Upon surpassing a specific threshold, the mutation level of mtDNA often induces phenotypic changes within cells, thereby leading to alterations in mitochondrial function and signal transduction.

PC tissues harbor extensive somatic mutations in mitochondrial DNA (mtDNA). Somatic mutations occur at high frequency in the mtDNA control region (D-loop) in up to 90% of PC samples, indicating that mitochondrial mutations represent an early event in prostate carcinogenesis (Chen et al., 2002). Whole-genome sequencing studies further confirm that PC patients carry an average of one mtDNA single nucleotide variant (mtSNV), and the mutational burden is positively correlated with disease aggressiveness (Hopkins et al., 2017). Notably, bone metastasis sites exhibit a significantly higher number of mtDNA mutations compared to primary tumors or soft tissue metastases, suggesting that the bone microenvironment exerts strong selective pressure on the mitochondrial genome (Arnold et al., 2015). For example, the missense mutation at nucleotide position 10,398 (A10398G), resulting in a Thr114Ala substitution in the ND3 protein, was specifically detected in bone metastases of 7 out of 10 advanced PC patients, but not in matched primary tumors or soft tissue metastases (Keith et al., 2015).

mtDNA copy number (mtDNA-CN) is highly heterogeneous in PC. Multiple studies have reported significantly reduced mtDNA-CN in prostate tumor tissues compared to adjacent normal tissues (Kalsbeek et al., 2018). However, other studies have observed increased mtDNA-CN in subsets of tumors, with higher copy numbers associated with adverse pathological features, including advanced stage, extracapsular extension, and a trend toward higher Gleason scores (Kalsbeek et al., 2018). This heterogeneity reflects the genomic instability of PC, and alterations in mtDNA-CN may exhibit race-specific patterns: elevated mtDNA-CN is associated with increased PC risk in White patients, but no such association has been observed in Black patients (Flores et al., 2025). Additionally, levels of circulating cell-free mtDNA (ccf-mtDNA) are significantly elevated in PC patients and correlate with tumor burden and poor prognosis (Ellinger et al., 2008; Borah et al., 2023).

Large-scale mtDNA deletions are frequently observed in PC. The 3.4 kb deletion (3.4 kbΔ) has been proposed as a biomarker detectable in both tissue biopsies and urine samples from PC patients. Combining the detection of this deletion with mtDNA-CN measurement improves diagnostic accuracy for PC (Maragh et al., 2015). Another common deletion, the 4977 bp deletion (mtDNA4977), is detected at significantly higher rates in PC tissues compared to benign prostatic hyperplasia tissues and is independently associated with higher Gleason scores (Yu and Yan, 2010).

### 3.2 Role of mtDNA in PC

mtDNA mutations can lead to excessive generation of ROS. Cybrids of the PC3 cell line carrying the pathogenic ATP6 T8993G mutation formed tumors in nude mice with volumes seven times larger than those generated by wild-type cybrids and produced significantly higher levels of ROS (Petros et al., 2005). Elevated ROS can further damage both nuclear and mitochondrial DNA, creating a vicious cycle that accelerates tumor progression (Chen and Kadlubar, 2004). The loss or mutation of mtDNA can confer resistance to apoptosis. Studies have shown that mtDNA-depleted LNCaP cells acquire the ability to grow in an androgen-independent manner and can form tumors in castrated mice. Reconstitution with normal mtDNA restores androgen dependency (Higuchi et al., 2006). This mechanism involves the activation of the PI3K/Akt2 signaling pathway: in mtDNA-depleted cells, activated Akt2 phosphorylates downstream substrates (such as GSK3β, c-Myc, and MMP-9), thereby inhibiting anoikis, enhancing migratory capacity, and promoting glycolytic metabolism (Moro et al., 2009). Furthermore, mtDNA depletion can activate the calcineurin/PI3K/AKT signaling axis, upregulate the expression of miR-1245 and Skp2, and suppress the translation of BRCA2 protein. This impairs homologous recombination (HR) repair and sensitizes cells to PARP inhibitors such as rucaparib (Arbini et al., 2013). mtDNA mutations can exacerbate metabolic dysregulation by disrupting respiratory chain complex function. For instance, the recurrent m.6267G>A mutation (resulting in an Ala122Thr substitution in MT-CO1) impairs cytochrome c oxidase activity, reduces respiratory capacity, and promotes PC growth (Gallardo et al., 2006).

The expression level of mtDNA-encoded cytochrome oxidase I was decreased in PC tissue slices, and PC cancer cell lines exhibited reduced mtDNA content compared to normal cells (Moro et al., 2009). Mechanistically, the decrease in mtDNA levels in PC cells can promote resistance to apoptosis and invasion through the activation of the PI3K/Akt2 pathway, suggesting a causal relationship between decreased mtDNA content and the development and invasiveness of PC (Moro et al., 2009). Mora et al. also discovered that highly invasive and androgen-independent PC-3 cells, as well as androgen-independent DU145 and C4-2 cells, exhibited lower mtDNA content compared to androgen-dependent LNCaP cells (Moro et al., 2008). In PC-3 cells, the depletion of mtDNA was accompanied by a decrease in mitochondrial membrane potential, increased resistance to apoptosis, and reduced sensitivity to paclitaxel treatment (Moro et al., 2008). Consistent with the aforementioned findings, Higuchi et al. demonstrated that the depletion of mtDNA could induce the transition of androgen-dependent LNCaP cells to androgen-independent status, increase cell migration, and confer resistance to common chemotherapeutic drugs (Higuchi et al., 2006). Moreover, the restoration of mitochondrial DNA clones reversed the dependence of PC cells on androgens (Higuchi et al., 2006).

In TME, senescent tumor cells release mtDNA, which is packaged into extracellular vesicles and selectively transferred to polymorphonuclear myeloid-derived suppressor cells (PMN-MDSCs). This transfer enhances the immunosuppressive activity of PMN-MDSCs via the cGAS–STING–NF-κB signaling pathway, thereby promoting tumor progression (Lai et al., 2025; Canadas et al., 2025). The release of mtDNA is mediated by the voltage-dependent anion channel (VDAC). Pharmacological inhibition of VDAC reduces extracellular mtDNA levels, reverses PMN-MDSC-driven immunosuppression, and enhances chemotherapy efficacy (Lai et al., 2025). These findings suggest that targeting mtDNA release may remodel the immunosuppressive tumor microenvironment and improve treatment outcomes.

In summary, mtDNA plays an important role in the development of PC, influencing cancer occurrence and progression. Further research will contribute to a deeper understanding of the pathogenesis of PC and provide a theoretical basis for the development of related therapeutic strategies.

### 3.3 Diagnostic and prognostic biomarkers

The cumulative burden of acquired mtDNA variations may serve as an oncogenic biomarker in prostate cancer. For example, the MT-ND4 m.10398A>G allele is frequently detected in bone metastases and demonstrates a significant positive correlation with Gleason score at diagnosis and recurrence (Francis et al., 2013). The detectability of mtDNA alterations in body fluids makes them promising targets for liquid biopsy. Plasma levels of circulating cell-free mtDNA (ccf-mtDNA) are significantly elevated in prostate cancer patients and are independent of total PSA levels (Ellinger et al., 2008). Detection of the 3.4-kb deletion (3.4 kbΔ) in urine shows high specificity for prostate cancer, and its combination with mtDNA copy number (mtDNA-CN) further improves diagnostic accuracy (Maragh et al., 2015). Additionally, somatic mtDNA mutational load is significantly associated with higher Gleason scores and biochemical recurrence, suggesting its potential as a prognostic indicator (Kalsbeek et al., 2016). Circulating mtDNA levels are closely associated with patient survival. Elevated plasma levels of mtDNA and mtRNA in advanced prostate cancer patients correlate with poorer 2-year survival rates, with high mtRNA levels serving as an independent predictor of overall survival (Mehra et al., 2007). Patients with serum short-fragment mtDNA (79 bp) levels above the 75th percentile have a significantly increased risk of PSA progression, representing the strongest predictor in multivariate analysis (Ellinger et al., 2008).

### 3.4 Targeting strategies

Disruption of mitochondrial replication or transcription mechanisms leads to mitochondrial dysfunction, resulting in energy insufficiency, growth inhibition, senescence, or even apoptosis (Sharma and Sampath, 2019). Due to the lack of histone protection and limited repair capacity, mtDNA becomes an attractive target for tumor treatment (Xiao et al., 2018). Therefore, there is an increasing number of anticancer drugs and treatment methods targeting mitochondria and mtDNA under development (Table 2).

**TABLE 2:** Targeting mtDNA in PC.

Targeted drug or treatment	Primary mechanism	Current limitations/Challenges	Translational readiness	Ref.
Pentamidine	Inhibits mtDNA transcription, leading to mtDNA depletion and mitochondrial dysfunction	May be ineffective against PC cells with inherently low mtDNA content; drug repurposing requires reassessment of anticancer efficacy and safety	Preclinical research	Liu et al. (2020)
IR (99mTc-TPP-BBN)	Cause localized mtDNA damage via targeted delivery	Involves complex drug delivery systems; challenges with tissue penetration depth of radiation/light and targeting efficiency	Preclinical research	Fernandes et al. (2022)
PDT ([1a] Cl and [3a] Cl)				Echevarria et al. (2022)

Abbreviations: PC: prostate cancer; IR: ionizing radiation; PDT: photodynamic therapy.

Pentamidine is a cationic aromatic diamidine drug that has been clinically used for decades to treat parasitic diseases such as African trypanosomiasis and leishmaniasis (Pearson and Hewlett, 1985; Zhang et al., 2022). Liu et al. found that after treatment with Pentamidine, the transcription levels of the majority of mtDNA significantly decreased (Liu et al., 2020). The degree of mtDNA reduction induced by Pentamidine was highly correlated with the differential sensitivity of PC cell lines to Pentamidine, suggesting that Pentamidine may inhibit PC by targeting mtDNA (Liu et al., 2020). In PC, Pentamidine can induce mtDNA reduction, morphological changes in mitochondria, and mitochondrial dysfunction, leading to cancer cell apoptosis (Liu et al., 2020). Moreover, in a xenograft mouse model, Pentamidine exhibited tumor-suppressive effects on the growth and metastasis of PC (Liu et al., 2020). This suggests that Pentamidine can serve as an effective mtDNA-targeting drug to inhibit the progression of PC.

Ionizing radiation (IR) is an effective and commonly used cancer treatment method, primarily relying on DNA damage for tumor suppression (Santivasi and Xia, 2014). Mitochondria have emerged as an intriguing extranuclear target for IR-based cancer therapy (Averbeck and Rodriguez-Lafrasse, 2021). Fernandes et al. designed a dual-targeting system, 99mTc-TPP-BBN, based on the single-targeting homologue 99mTc-BBN, which exhibits enhanced selective uptake by PC cells and accumulation in mitochondria (Fernandes et al., 2022). Compared to 99mTc-BBN, 99mTc-TPP-BBN effectively inhibits the activity of PC cells, and this effect is associated with a decrease in mtDNA copy number (Fernandes et al., 2022). Photodynamic therapy (PDT) is a non-invasive treatment method used for cancer therapy (Jia et al., 2023). Echevarría et al. developed Ir(III) biscyclometalated complexes, [1a] Cl and [3a] Cl, for PDT in PC (Echevarria et al., 2022). Mechanistically, [1a] Cl and [3a] Cl may induce mtDNA damage and mitochondrial membrane depolarization through a photochemical oxidation mechanism (Echevarria et al., 2022). These findings suggest the potential of targeting mtDNA as a therapeutic approach in PC treatment.

It is worth noting that the characteristic of low mtDNA content in PC may hinder current mtDNA-targeted therapeutic strategies (Moro et al., 2009; Moro et al., 2008; Higuchi et al., 2006). As demonstrated by the study conducted by Higuchi et al., the reconstruction of mtDNA in PC may enhance the effectiveness of current mtDNA-targeted therapeutic strategies (Higuchi et al., 2006).

### 3.5 mtDNA in PC: challenges from biomarker to therapeutic target

In PC, mtDNA demonstrates considerable diagnostic and prognostic potential. However, its functional characterization and therapeutic targeting remain challenging. Apparent contradictions emerge in its application as a biomarker: mtDNA copy number can be either decreased or increased in PC tissues, likely reflecting tumor heterogeneity, disease progression, or ethnic differences (Kalsbeek et al., 2018). Although circulating cell-free mtDNA shows promise for non-invasive diagnosis (Francis et al., 2013; Maragh et al., 2015; Kalsbeek et al., 2016; Mehra et al., 2007; Ellinger et al., 2008), its release mechanisms and the biological significance of fragment sizes remain poorly defined.

At the functional level, a major knowledge gap lies in distinguishing whether mtDNA mutations act as oncogenic “drivers” or merely represent passenger events. While certain mutations—such as A10398G in bone metastases—exhibit strong signals of positive selection (Keith et al., 2015), the functional impact of most mtDNA variants remains inadequately validated. Furthermore, a significant therapeutic paradox also arises in targeting mtDNA: many aggressive PC cells inherently exhibit low mtDNA content, which may confer intrinsic resistance to agents designed to deplete or damage mtDNA, such as pentamidine (Kalsbeek et al., 2018; Liu et al., 2020). Therefore, future therapeutic strategies may require combination approaches—for instance, first sensitizing cancer cells to mitochondrial dependence using other means, followed by mtDNA-targeted treatment.

Moving forward, larger cohort studies are needed to validate the clinical utility of specific mtDNA mutations or copy number alterations. Therapeutically, exploring synergies between mtDNA-targeting agents and drugs that induce metabolic stress (e.g., OXPHOS inhibitors) may offer a viable strategy to overcome the current limitations of mtDNA-directed therapy.

## 4 Overview of mitochondrial ROS in PC

### 4.1 Role of mitochondrial ROS in PC

ROS are primarily generated as byproducts of mitochondrial respiration, and their dysregulated production is a hallmark of cancer cells (Tossetta et al., 2023; Afzal et al., 2023). In PC, ROS levels are intrinsically higher than in normal cells, driven by mechanisms such as the hyperactivation of mitochondrial glycerol-3-phosphate dehydrogenase (mGPDH) and NADPH oxidase (NOX) (Kumar et al., 2008; Chowdhury et al., 2007).

These elevated ROS play a context-dependent, dual role in tumor progression. At moderate levels, ROS function as signaling molecules that promote proliferative and pro-survival pathways (Joshi et al., 2020; Petros et al., 2005). However, excessive ROS induce oxidative stress, leading to macromolecular damage—including protein misfolding, lipid peroxidation, and DNA lesions—and ultimately activate intrinsic apoptosis through mechanisms involving Bcl-2 family proteins, mitochondrial membrane depolarization, and JNK/MAPK signaling (Nandha et al., 2025; Shao et al., 2021; Yan et al., 2013).

The therapeutic targeting of this redox balance, however, is complex. While epidemiological data and the increased oxidative stress associated with aging suggest a value for antioxidants in PC prevention (Perez-Gomez et al., 2023), clinical evidence has been largely inconsistent. The large-scale SELECT trial, for instance, found that supplementation with selenium and vitamin E not only failed to prevent PC but potentially increased its risk in some individuals (Klein et al., 2001). This paradox may be explained by the stage-specific functions of ROS. Most studies on oxidative stress-induced carcinogenesis focus on late-stage PC models, emphasizing the later steps of cancer development (Marzioni et al., 2023; Kalinina et al., 2022). Contrary to the pro-tumorigenic role of oxidative stress in advanced PC, evidence suggests that ROS may exert tumor-suppressive effects during early tumorigenesis. For example, inhibition of ROS was shown to suppress the proliferation of premalignant prostate epithelial cells in a mouse model, indicating a protective role of ROS in initial stages (Martinez et al., 2012). This functional heterogeneity underscores the challenge of broad antioxidant intervention.

Despite the ambiguities surrounding chemoprevention, the susceptibility of PC cells to oxidative damage remains a compelling therapeutic avenue. Further investigation into precisely targeting the mitochondrial redox state to induce lethal levels of oxidative stress represents a crucial and promising direction for PC treatment.

### 4.2 Targeting strategies

The induction of excessive ROS accumulation in cells represents a fundamental mechanism of action for certain preclinical models of PC treatment drugs, including histone deacetylase (HDAC) inhibitors, cyclin-dependent kinase 4/6 inhibitors, and natural product extracts (Table 4).

#### 4.2.1 Drugs

HDAC inhibitors are a novel class of anticancer drugs that can inhibit the proliferation and induce apoptosis of various cancer cells (Ho et al., 2020). HDAC inhibitor MHY4381 induced apoptosis in PC cells by inducing ROS production (Richa et al., 2020). The development of PC is also closely related to the action of neuroendocrine products. Furthermore, cyclin-dependent kinase 4/6 inhibitor abemaciclib can induce apoptosis in metastatic castration-resistant PC (mCRPC) AR negative PC-3 and AR mutant LNCaP PC cells by inducing ROS production (Guney Eskiler et al., 2022).

The accumulation of mitochondrial ROS is also associated with specific forms of cell death in PC. For instance, CRPC is frequently accompanied by mitochondrial metabolic reprogramming. Androgen signaling promotes cell proliferation via p66Shc-mediated mitochondrial ROS production (Veeramani et al., 2008). The mitochondria-targeted melatonin analogue (Mito-Mel) induces pyroptosis in CRPC cells and promotes an immune response by increasing ROS generation (Chen et al., 2025). Furthermore, SREBF1-mediated metabolic reprogramming is linked to ferroptosis resistance in CRPC. Elevated transcriptional activity of SREBF1 enhances fatty acid and cholesterol metabolism, conferring resistance to ferroptosis through multiple mechanisms. Treatment with the SREBF1 inhibitor betulin significantly promotes ROS accumulation, glutathione (GSH) depletion, thereby increasing chemosensitivity (Wei et al., 2025). Similarly, dihydroartemisinin, when combined with radiotherapy or chemotherapy, enhances the therapeutic effect on PC by promoting ROS accumulation and inducing ferroptosis (Guan et al., 2024).

#### 4.2.2 Natural product

Additionally, some natural product extracts can inhibit the progression of PC by inducing ROS production. Wogonin, a flavonoid compound isolated from Scutellaria root, can dose- and time-dependently induce ROS production and apoptosis in human cancer cells (Banik et al., 2022). In PC, wogonin promotes fatty acid synthesis and oxidation through the AKT-SREBP1-FASN pathway, further generating excessive ROS, thereby inducing cell apoptosis (Sun et al., 2022). Fructus Amomi is a volatile oil widely used in traditional medical systems in multiple countries (Nogueira et al., 2020). H. spicatum essential oil, derived from Fructus Amomi, inhibits the progression of PC by inducing the accumulation of ROS (Ray et al., 2023). Aeroplysinin-1, a brominated isoxazole alkaloid isolated from a marine sponge species, exerts cytotoxic effects on PC mainly through ROS-induced mitochondria-dependent apoptosis (Garcia-Vilas et al., 2015). Similar to Aeroplysinin-1, Berbamine, Neferine, Dieckol, Brassinin, mannose, myricetin, fisetin and 11-epi-artapshin can promote apoptosis of PC cells and inhibit cancer progression by inducing the accumulation of excessive ROS (Zhao et al., 2023; Dasari et al., 2023; Karuppaiya et al., 2023; Deng et al., 2022; Fattahian et al., 2022; Kwon et al., 2023; Kaleem et al., 2016).

#### 4.2.3 Treatment methods

The induction of excessive ROS accumulation in cells is also a core mechanism of some PC treatment methods, including PDT, IR, nanocarrier therapy, and Sonodynamic Therapy (SDT) (Bolitho et al., 2020; Tabatabaie et al., 2022; Wu et al., 2020; Turkkol et al., 2025). PDT is a non-invasive treatment method used for cancer therapy (Jia et al., 2023). PDT utilizes spatially targeted visible light (λ = 500–800 nm) to irradiate photosensitizer prodrugs, which are non-toxic in the dark (Jia et al., 2023). Irradiation of the compounds with single-photon blue light or two-photon red light generates high concentrations of ROS, particularly singlet oxygen (_1_O^2^), in a highly localized manner within the irradiated area, subsequently inducing apoptosis in the tumor site (Jia et al., 2023). Therefore, photodynamic therapy can help reduce the unnecessary side effects typically experienced by patients undergoing other chemotherapy treatments. PC is a strong candidate for PDT treatment. Bolitho et al. used iridium photosensitizers-dependent PDT to cause extensive and specific changes in mitochondria, leading to the killing of PC cells (Bolitho et al., 2020). Additionally, IR is an effective and commonly used cancer treatment method (Santivasi and Xia, 2014). Gold nanoparticles can enhance the sensitivity of tumor cells to radiation damage, thereby improving the efficacy of anticancer drugs (Tabatabaie et al., 2022). Mechanistically, the addition of gold nanoparticles enhances the level of ROS production in PC cells after radiotherapy, increasing the cytotoxicity of radiotherapy to cancer cells (Tabatabaie et al., 2022). Furthermore, some novel targeted strategies based on the characteristics of PC have been validated in preclinical models. Due to the unique metabolism of PC, the tumor microenvironment exhibits lower pH compared to normal tissues (Ma et al., 2023). Based on this unique characteristic, Wu et al. developed a mitochondrial oxidative stress amplifier by exploiting pH differences (Wu et al., 2020). Wu et al. effectively delivered calcium oxide to the tumor site using nanocarriers and released ROS, which activated mitochondria-mediated apoptosis both *in vitro* and *in vivo*, exhibiting excellent anti-tumor effects (Wu et al., 2020). Specifically, the non-cytotoxic free calcium oxide can release ROS responsively under acidic tumor microenvironment, inducing significant apoptosis through mitochondria-mediated oxidative stress while minimizing damage to normal tissues, thus achieving superior anti-tumor efficacy (Wu et al., 2020). SDT represents another non-invasive treatment approach that utilizes ultrasound to activate sensitizing agents and induce cancer cell death (Turkkol et al., 2025). Studies have demonstrated that optimizing ultrasound parameters to increase cavitation density can enhance the efficacy of Ce6-mediated SDT (Turkkol et al., 2025). This leads to elevated ROS generation, loss of mitochondrial membrane potential, and disruption of the mitochondrial unfolded protein response (Turkkol et al., 2025). These findings provide a promising new direction for the local treatment of PC.

### 4.3 The dual role of ROS: a therapeutic dilemma

The role of ROS in PC is characteristically dual-edged, which helps explain why direct antioxidant-based prevention strategies, such as those in the SELECT trial, have largely failed (Klein et al., 2001). The central paradox in this field stems from the context-dependent nature of ROS—its effects are determined by both concentration and disease stage. At low levels, ROS function as signaling molecules that promote proliferation and survival (Joshi et al., 2020; Petros et al., 2005), whereas excessive ROS cause irreversible damage and trigger cell death (Nandha et al., 2025; Shao et al., 2021; Yan et al., 2013). Moreover, ROS may act as tumor suppressors during early carcinogenesis but drive progression in advanced disease (Joshi et al., 2020; Petros et al., 2005; Nandha et al., 2025; Shao et al., 2021; Yan et al., 2013). This complexity renders global “ROS reduction” an oversimplified and potentially counterproductive strategy.

Successful therapeutic paradigms have shifted from indiscriminate antioxidant use to the strategic application of ROS-inducing agents that elevate intracellular ROS beyond a lethal threshold. This class includes conventional modalities such as radiotherapy and chemotherapy, as well as emerging approaches like PDT, SDT, and numerous natural compounds (Table 3). These treatments share a common mechanism: inducing severe oxidative stress that activates various forms of programmed cell death, including apoptosis, pyroptosis, and ferroptosis.

**TABLE 3:** Targeting ROS in PC.

Targeted drug or treatment	Primary mechanism	Current limitations/Challenges	Translational readiness	Ref.
HDAC inhibitors (MHY4381)	Disrupt specific signaling pathways, leading to ROS accumulation and apoptosis induction	Potential effects on normal cells, lack of tumor selectivity; prone to inducing compensatory mechanisms	Some drugs in clinical trials, but their pro-oxidant anticancer mechanism is often a recent discovery	Richa et al. (2020)
Cyclin-dependent kinase 4/6 inhibitor (abemaciclib)				Guney Eskiler et al. (2022)
Mito-Mel	Targets mitochondria, suppresses metabolism, and induces pyroptosis	Novel compound, insufficient pharmacokinetic and toxicity data	Preclinical research	Chen et al. (2025)
Betulin	Inhibits lipid metabolism, sensitizing cells to ferroptosis, accompanied by ROS accumulation	Complex lipid metabolism pathways, prone to resistance; may disrupt normal lipid homeostasis	Preclinical research	Wei et al. (2025)
dihydroartemisinin combined with radiotherapy or chemotherapy	Promotes ROS accumulation and induces ferroptosis; exhibits synergistic effects with conventional therapies	Optimal sequencing and dosing with combination partners need clarification; potential for increased normal tissue toxicity during combination therapy	Preclinical research (Dihydroartemisinin itself is clinically approved for malaria)	Guan et al. (2024)
Natural product extracts (Wogonin, H. spicatum essential oil, Aeroplysinin-1, Berbamine, Neferine, Dieckol, Brassinin, mannose, 11-epi-artapshin)	Induce mitochondrial dysfunction and ROS burst via multiple pathways	Complex composition, unclear precise targets; generally low bioavailability	Predominantly preclinical research	Sun et al. (2022), Ray et al. (2023), Garcia-Vilas et al. (2015), Zhao et al. (2023), Dasari et al. (2023), Karuppaiya et al. (2023), Deng et al. (2022), Fattahian et al. (2022), Kwon et al. (2023), Kaleem et al. (2016)
PDT (iridium photosensitizers)	Generate high local concentrations of ROS in tumors via external energy (light/sound) or targeted delivery	Equipment-dependent; limited tissue penetration depth; long-term biosafety of nanomaterials requires assessment	PDT, Radiotherapy clinically applied; novel nanocarriers and SDT in preclinical research	Bolitho et al. (2020)
IR (Gold nanoparticles)				Tabatabaie et al. (2022)
Nanocarriers delivering (calcium oxide)				Wu et al. (2020)
SDT (Ce6)				Turkkol et al. (2025)

Abbreviations: ROS: reactive oxygen species; HDAC: histone deacetylase; PC: prostate cancer; mCRPC: metastatic castration-resistant PC; Mito-Mel: mitochondria-targeted melatonin analogue; IR: ionizing radiation; PDT: photodynamic therapy; SDT: sonodynamic therapy.

A primary challenge in this area is achieving tumor selectivity. Ensuring that ROS-inducing agents generate cytotoxic ROS levels specifically in cancer cells—while sparing normal tissues—requires advanced targeting strategies. One promising direction involves designing delivery systems (e.g., nanocarriers) that exploit PC-specific metabolic traits, such as the acidic tumor microenvironment. Additionally, a deeper understanding of the specific ROS species generated by different therapies and their corresponding downstream death signaling pathways will be essential for rational combination therapy design.

## 5 Overview of MOMP and apoptosis-related proteins in PC

### 5.1 Role of MOMP and apoptosis-related proteins in PC cell death

Mitochondria play a central role in programmed cell death by controlling apoptotic pathways within cells (Desagher and Martinou, 2000). Among them, MOMP and the release of cytochrome c are critical processes in apoptosis (Karch et al., 2015). MOMP is closely associated with the mitochondrial permeability transition pore (MPTP) (Karch et al., 2015). Oxidative stress and activation of pro-apoptotic proteins can lead to the opening of MPTP, triggering MOMP (Zhu et al., 2018; Jayappa et al., 2023).

During mitochondrial apoptosis, MOMP is the first crucial step mediated by Bcl-2 family proteins (Jayappa et al., 2023). In the absence of apoptotic stimuli, anti-apoptotic proteins Bcl-2, Bcl-xl, and MCL1 form heterodimers with pro-apoptotic proteins Bax or Bak, inhibiting the pro-apoptotic activity of Bax/Bak and maintaining the integrity of the mitochondrial outer membrane, thus blocking mitochondrial apoptosis (Desagher and Martinou, 2000). However, in the presence of apoptotic stimuli, the expression of pro-apoptotic protein Bax increases, or the expression of proteins containing only the BH3 domain (such as Bad, Bid, and Bim) increases. They competitively bind to anti-apoptotic protein Bcl-2, releasing the inhibited Bax/Bak (Desagher and Martinou, 2000). The free Bax and Bak form oligomers, leading to MOMP and directly releasing apoptotic factors such as cytochrome c into the cytoplasm (Sheridan et al., 2008). When cytochrome c binds to apoptosis protease-activating factor 1 (APAF-1) on the cell membrane, an apoptosome is formed, further activating procaspase-9 and cleaving it into its active form (Elena-Real et al., 2018). Caspase-9 acts as an initiator of the mitochondrial apoptosis pathway, promoting downstream activation of caspases (McComb et al., 2019). Caspases-3 and -7, activated by caspase-9, then initiate a cascade reaction that leads to apoptosis in cancer cells (McComb et al., 2019). In addition to these processes, BH3-only proteins can also initiate mitochondria-related cell apoptosis by activating Bcl-xl and MCL1 (Huang et al., 2019). Changes in the interaction between members of the Bcl-2 family may result in decreased mitochondrial outer membrane permeability, thereby disrupting mitochondrial integrity (Bruckheimer et al., 1998). Therefore, the balance between anti-apoptotic and pro-apoptotic proteins of the Bcl-2 family, as well as the activation of caspases, is essential for the regulation and execution of cell apoptosis. However, in PC, the expression of anti-apoptotic proteins Bcl-2, Bcl-xl, and MCL1 is enhanced, which contributes to its strong chemoresistance (Doroshenko et al., 2022). Therefore, further exploration of the mechanisms of mitochondria in PC development and treatment, particularly regarding MOMP and the regulation of Bcl-2 family proteins, is necessary.

### 5.2 Targeting strategies

**FIGURE 2:**
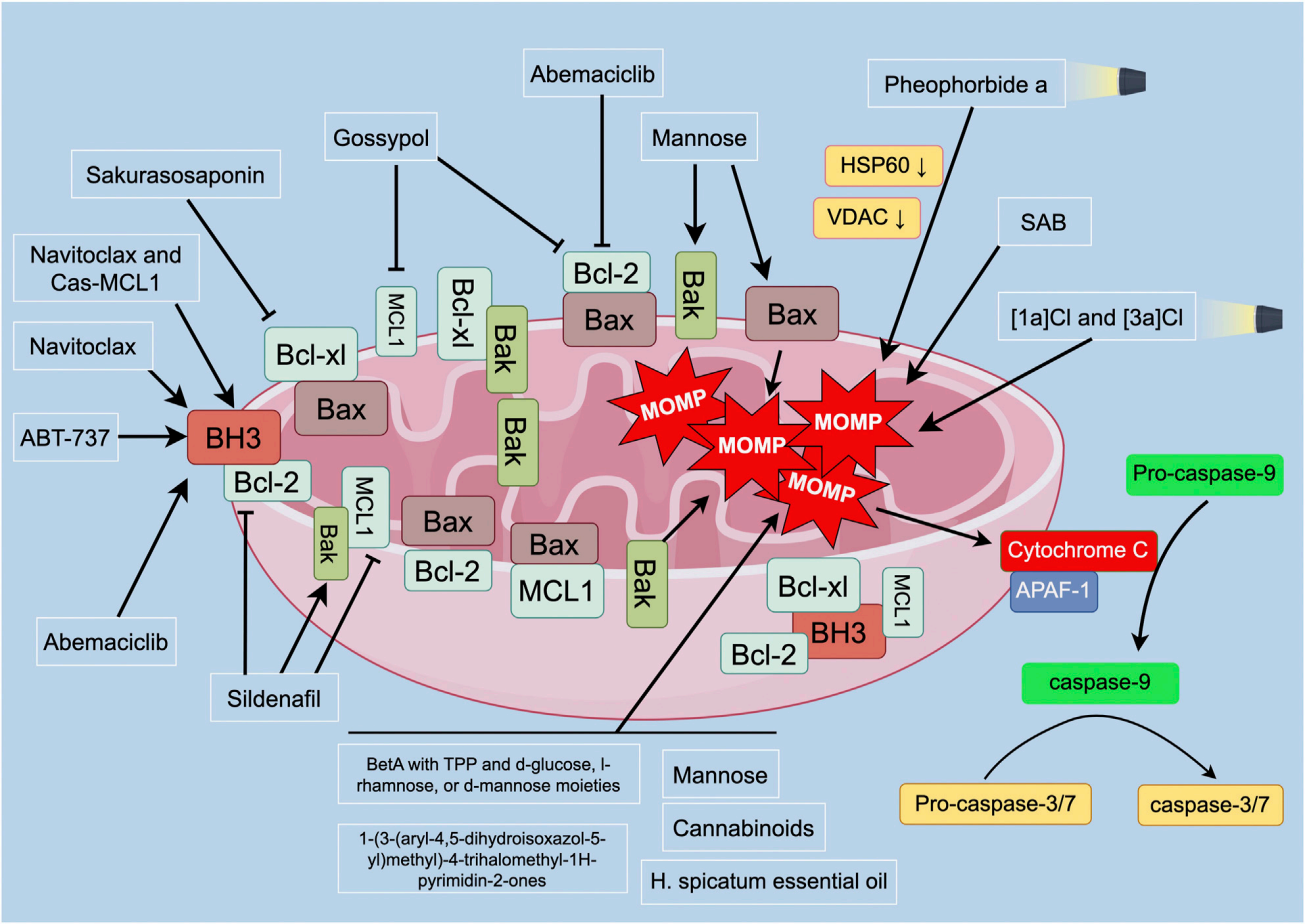
Overview of MOMP and apoptosis-related proteins in PC. Mitochondria control apoptotic pathways in programmed cell death. MOMP and cytochrome c release are critical in apoptosis. Anti-apoptotic proteins inhibit Bax/Bak, maintaining mitochondrial integrity. In the presence of apoptotic stimuli, Bax is released, leading to MOMP and cytochrome c release. Cytochrome c binds to APAF-1, activating caspase-9. Caspase-9 initiates the mitochondrial apoptosis pathway, activating downstream caspases (caspase-3 and -7), inducing apoptosis. BH3-only proteins can also initiate mitochondria-related cell apoptosis by activating Bcl-xl and MCL1. Disruption of the balance between anti-apoptotic and pro-apoptotic proteins affects mitochondrial outer membrane permeability and integrity. Targeting strategies for inducing apoptosis in PC cells include iridium photosensitizers, PDT, betulinic acid derivatives, synthetic compounds, and natural products. These strategies can affect MOMP, induce mitochondrial depolarization, and promote apoptosis in PC cells. Combination therapies targeting anti-apoptotic proteins have also shown promising anticancer effects in PC treatment. Abbreviations: BH3: Bcl-2 homology domain 3; Bcl-xl: B-cell lymphoma-extra large; Bcl-2: B-cell lymphoma 2; MCL1: myeloid cell leukemia 1; MOMP: mitochondrial outer membrane permeabilization; Bax: Bcl-2-associated X protein; Bak: Bcl-2 homologous antagonist/killer; APAF-1: apoptotic protease activating factor 1; HSP60: heat shock protein 60; VDAC: voltage-dependent anion channel: SAB: 6-S-(1,4-naphthoquinone-2-yl)-d-glucose chimera molecule.

PC exhibits strong proliferation activity and resistance to treatment due to the high expression of anti-apoptotic proteins such as Bcl-2, Bcl-xl, and MCL1 (Doroshenko et al., 2022). Therefore, in addition to targeting MOMP, some compounds or treatment methods can exert anticancer effects by targeting anti-apoptotic proteins or acting on both MOMP and anti-apoptotic proteins. Strategies such as photodynamic therapy (PDT), natural products, and synthetic compounds have shown potential therapeutic effects in regulating mitochondrial function and apoptotic pathways (Figure 2; Table 4).

**TABLE 4:** Targeting MOMP and anti-apoptotic proteins in PC.

Targeted drug or treatment	Primary mechanism	Current limitations/Challenges	Translational readiness	Ref.
Sakurasosaponin	Downregulate anti-apoptotic proteins (e.g., Bcl-xL) and/or induce mitochondrial depolarization	Multi-target effects, specific molecular mechanisms unclear; efficacy and stability need optimization	Preclinical research	Song et al. (2020)
Mannose, fructus Amomi essential oil, cannabinoids			Preclinical research	Ray et al. (2023), Shoeib et al. (2022), Deng et al. (2022)
Hypericin, Gossypol	Inhibit Bcl-2 and/or MCL1	Low selectivity, potential off-target effects; Gossypol has known toxicity	Preclinical research	Doroshenko et al. (2022)
BetA conjugates with TPP and d-glucose	Directly induce mitochondrial depolarization, cytochrome c release, and caspase activation	Mostly newly designed molecules; lack comprehensive preclinical pharmacology and toxicology assessment	Preclinical research	Tsepaeva et al. (2023)
Compound 9c				Schmauss et al. (1988)
SAB				Dyshlovoy et al. (2020)
Anti-apoptotic protein inhibitors (ABT-737, Navitoclax)	Inhibits Bcl-2, Bcl-xL, Bcl-w	Dose-limiting thrombocytopenia due to Bcl-xL inhibition; limited efficacy as monotherapy in solid tumors	In clinical trials for various cancers	Oltersdorf et al. (2005), Tse et al. (2008)
MET	Induces mitochondrial calcium overload and alters membrane protein expression, leading to apoptosis	As a serotonin receptor antagonist, may have off-target neurological effects; mechanism in PC requires further validation	Preclinical research	Yang et al. (2020)
Cyclin-dependent kinase 4/6 inhibitor (abemaciclib)	Induces mitochondrial depolarization and inhibits PC progression; induces apoptosis via increased ROS production in mCRPC cells	May exhibit limited efficacy in certain PC subtypes; optimal patient selection criteria undefined	Clinical stage (approved for other cancers), investigation in PC ongoing	Guney Eskiler et al. (2022)
Sildenafil	Enhances chemotherapy-induced mitochondrial damage and downregulation of MCL1/Bcl-2	Combination strategy; optimal dosing and timing need refinement	Preclinical research (Sildenafil is approved)	Hsu et al. (2020)
PDT (Pheophorbide a)	Light activation downregulates MOMP-associated proteins (e.g., VDAC), disrupting membrane integrity	Limited by tissue penetration depth; suitable only for superficial or endoscopically accessible tumors	Some PSs clinically approved; novel PSs under development	Xu et al. (2020)
PDT Ir(III) biscyclometalated complexes ([1a]Cl and [3a]Cl)				Echevarria et al. (2022)

Abbreviations: MOMP: mitochondrial membrane permeabilization; PC: prostate cancer; BetA: betulinic acid; Bcl-xl: B-cell lymphoma-extra large; Bcl-2: B-cell lymphoma 2; MCL1: myeloid cell leukemia 1; Bax: Bcl-2-associated X protein; Bak: Bcl-2, homologous antagonist/killer; Bcl-w: B-cell lymphoma-w; SAB: 6-S-(1,4-naphthoquinone-2-yl)-d-glucose chimera molecule; MET: methiothepin mesylate; PDT: photodynamic therapy.

#### 5.2.1 Natural product

The inhibition of AR activity through ADT is the standard treatment for metastatic PC, but tumors often recur (Rebello et al., 2021). Although patients initially respond to further AR inhibition, most experience recurrence within 1–2 years, which appears to be driven by multiple AR-dependent and independent mechanisms, possibly including increased expression of anti-apoptotic proteins (Desai et al., 2021). Therefore, targeting the AR signaling pathway may be an important therapeutic target for PC. The anti-apoptotic Bcl-2 family proteins, including Bcl-2, Bcl-xl, and MCL1, function by sequestering Bax and Bak and inhibiting BH3 (a pro-apoptotic protein that can activate Bax/Bak) (Mohammad et al., 2015). Song et al. discovered that a newly identified natural compound, sakurasosaponin, induces mitochondria-mediated cell death in androgen-dependent (LNCaP) and castration-resistant (22Rv1 and C4-2) PC cell lines (Song et al., 2020). Specifically, sakurasosaponin induces cell death by downregulating Bcl-xL expression and reducing mitochondrial membrane potential (Song et al., 2020). Fructus Amomi essential oil, cannabinoids, and mannose can inhibit the progression of PC by inducing mitochondrial depolarization (Ray et al., 2023; Shoeib et al., 2022; Deng et al., 2022). Additionally, mannose also enhances the expression of pro-apoptotic factors such as Bax and Bak (Deng et al., 2022). Hypericin and Gossypol are BH3 mimetics that inhibit anti-apoptotic Bcl-2 proteins and can inhibit the progression of PC (Doroshenko et al., 2022). Gossypol can interact with both Bcl-2 and MCL1, promoting apoptosis in PC cells (Doroshenko et al., 2022). In comparison, Hypericin acts only on Bcl-2 but exhibits stronger inhibitory effects on PC cells (Doroshenko et al., 2022). Hypericin and Gossypol have synergistic effects and may be combined with other BH3 mimetics targeting MCL1 or Bcl-xl proteins for combination therapy in PC (Doroshenko et al., 2022).

#### 5.2.2 Synthetic compounds

Several synthetic compounds have been shown to inhibit the progression of PC by targeting MOMP. Tsepaeva et al. developed a novel method to synthesize conjugates of biologically active lupane-type triterpenes with triphenylphosphine (TPP) and d-glucose, l-rhamnose, or d-mannose moieties (Tsepaeva et al., 2023). Using this method, they synthesized compounds based on betulinic acid (BetA) with TPP and d-glucose, which effectively downregulated mitochondrial membrane potential and induced apoptosis in PC cells (Tsepaeva et al., 2023). 9c, a synthetic1-(3-(aryl-4,5-dihydroisoxazol-5-yl)methyl)-4-trihalomethyl-1H-pyrimidin-2-one compound, could induce mitochondrial depolarization and activate the intrinsic pathway, leading to apoptosis in PC cells by increasing cleavage of caspase-9 and -3 (Schmauss et al., 1988). Dyshlovoy et al. designed and synthesized a novel 6-S-(1,4-naphthoquinone-2-yl)-d-glucose chimera molecule (SAB), which effectively induced apoptosis in human PC cells, including highly resistant cell lines (Dyshlovoy et al., 2020). SAB induced mitochondrial depolarization, release of cytotoxic mitochondrial proteins (AIF and cytochrome c) into the cytoplasm, upregulation of ROS, followed by activation of caspase-9 and -3, PARP cleavage, DNA fragmentation, and apoptosis (Dyshlovoy et al., 2020).

ABT-737 and Navitoclax (an oral analogue of ABT-737) are BH3 mimetics that directly bind to Bcl2, Bcl-xl, and Bcl-w (but not MCL1), blocking their interaction with pro-apoptotic protein BH3 and their ability to sequester Bax/Bak (Oltersdorf et al., 2005; Tse et al., 2008). Unfortunately, most solid tumors exhibit resistance to navitoclax and ABT-737 (Faber et al., 2015). This highlights the importance of inhibiting MCL1 expression or function in designing targeted mitochondrial therapies. Indeed, preclinical studies suggest that Navitoclax may be effective in PC treatment when MCL1 expression is reduced (Arai et al., 2018). Arai et al. reported that combining navitoclax (through Bcl-xl inhibition) with several kinase inhibitors (erlotinib, lapatinib, cabozantinib, sorafenib) induces rapid and significant apoptosis in PC cells after RNAi or CRISPR-mediated knockdown of MCL1 expression (Arai et al., 2018). Currently, BH3 mimetics targeting MCL1, including AMG176, S63845, and AZD5991, are becoming available and may have single-agent activity in certain subsets of tumors, but their efficacy in PC may still require combination therapy (Arai et al., 2020; Arai et al., 2018). Furthermore, the toxicity associated with direct MCL1 antagonists alone or in combination therapy remains to be determined.

#### 5.2.3 Drugs

Antagonists of serotonin receptors have been shown to inhibit the proliferation and induce cell death in PC, and this effect is mediated by targeting mitochondria (Yang et al., 2020). Methiothepin mesylate (MET), an antagonist of 5-HT1, induces mitochondrial calcium overload and changes in mitochondrial membrane protein expression, leading to apoptosis of PC cells (Yang et al., 2020). The cyclin-dependent kinase 4/6 inhibitor abemaciclib inhibits the progression of PC by inducing mitochondrial depolarization (Guney Eskiler et al., 2022). Particularly in mCRPC cell lines that are AR negative (PC-3) or have AR mutations (LNCaP), abemaciclib treatment induces apoptosis by increasing ROS production (Guney Eskiler et al., 2022). Sildenafil, a phosphodiesterase type 5 inhibitor, has been recognized for its cardioprotective and neuroprotective effects (Kukreja, 2013; Zinni et al., 2021). Hsu et al. found that sildenafil enhanced the therapeutic effect of vincristine in PC *in vitro* and *in vivo* (Hsu et al., 2020). Mechanistically, sildenafil enhanced vincristine-induced mitochondrial damage, including downregulation of MCL1, phosphorylation and downregulation of Bcl-2, upregulation of Bak, and loss of mitochondrial membrane potential, leading to apoptosis in PC cells (Hsu et al., 2020).

#### 5.2.4 Treatment methods

As mentioned earlier, iridium photosensitizers can induce apoptosis in PC cells by generating high concentrations of ROS, particularly singlet oxygen (_1_O^2^), specifically targeting mitochondria (Bolitho et al., 2020). Similarly, PDT can induce apoptosis in PC cells by affecting MOMP (Xu et al., 2020). Xu et al. found that after PDT treatment with Pheophorbide a, the expression of mitochondrial membrane proteins voltage-dependent anion channel (VDAC) and HSP60 was significantly downregulated, and the integrity of the mitochondrial membrane was compromised (Xu et al., 2020). In addition, Echevarría et al. developed Ir(III) biscyclometalated complexes, [1a]Cl and [3a]Cl, for PDT in PC (Echevarria et al., 2022). Mechanistically, [1a]Cl and [3a]Cl may induce mitochondrial depolarization through a photocatalytic oxidation mechanism, promoting apoptosis in PC cells (Echevarria et al., 2022). These findings suggest that interventions targeting proteins associated with MOMP could be a direction for photodynamic therapy, and further research will help us understand the role of mitochondrial integrity in the survival of PC cells.

### 5.3 Overcoming apoptotic resistance: combination strategies

PC cells establish robust defense mechanisms against mitochondrial apoptosis by upregulating anti-apoptotic proteins such as Bcl-2, Bcl-xL, and MCL1 (Doroshenko et al., 2022). A central paradigm in this field is that monotherapies targeting MOMP frequently prove ineffective, making combination strategies an imperative. The limited success of BH3 mimetics (e.g., ABT-737/Navitoclax) underscores the functional redundancy within the anti-apoptotic network—where inhibition of Bcl-2/Bcl-xL is often compensated by subsequent upregulation of MCL1 (Oltersdorf et al., 2005; Tse et al., 2008).

Consequently, the most effective approaches involve either concurrently targeting multiple anti-apoptotic proteins or combining BH3 mimetics with other treatment modalities (Arai et al., 2020; Arai et al., 2018). For example, Navitoclax demonstrates strong synergistic effects when used together with MCL1 inhibitors or certain kinase inhibitors (particularly under MCL1 knockdown conditions). Similarly, combining pro-apoptotic natural compounds (e.g., sakurasosaponin) or mitochondrial-disrupting synthetic agents with standard therapies can overcome the apoptotic threshold through multi-faceted mechanisms (Song et al., 2020; Hsu et al., 2020; Tsepaeva et al., 2023).

However, a significant translational challenge remains the current lack of reliable biomarkers to predict which patients will respond to specific BH3 mimetics or combination regimens. Future studies should focus on identifying such biomarkers to enable precision targeting of apoptotic pathways. In parallel, the *in vivo* toxicity profiles and therapeutic windows of direct anti-apoptotic proteins inhibitors require more comprehensive evaluation in prostate cancer models.

## 6 Overview of mitochondrial dynamics in PC

### 6.1 Role of mitochondrial dynamics in PC

Mitochondrial dynamics encompass mitochondrial biogenesis, fusion/fission, and mitophagy (Chan, 2020). Mitochondrial biogenesis is the generation of new mitochondria from pre-existing organelles (Grasso et al., 2020). It is regulated by stress signals and is associated with tumor cell proliferation, invasion, migration, and drug resistance (Grasso et al., 2020).

Mitochondrial fusion/fission refers to specific changes in mitochondrial morphology (Adebayo et al., 2021). Mitochondrial fusion, mediated by the outer membrane proteins MFN1 and MFN2 and the inner membrane protein OPA1, promotes mitochondrial network integrity, thereby facilitating content exchange and energy distribution (Baumgartner et al., 2024). In PC, the downregulation of fusion proteins such as OPA1 and MFN1 is frequently observed in advanced stages, further exacerbating mitochondrial dysfunction (Berger et al., 2025; Fontana et al., 2023). Functionally, alterations in complex I and enhanced mitochondrial fusion are associated with the progression of PC (Philley et al., 2016). Moreover, increased mitochondrial fusion is critical for chemotherapy sensitivity in drug-resistant tumor cells (Jin et al., 2022).

Mitochondrial fission is regulated by dynamin-related protein 1 (Drp-1) and its receptor proteins—such as FIS1, MTFP1, and MTFP2—leading to mitochondrial fragmentation (Youle and Karbowski, 2005). This process facilitates the removal of damaged mitochondria and enables adaptive responses to cellular energy demands. Excessive mitochondrial fission can trigger various types of mitochondria-mediated cell apoptosis and generate numerous mitochondrial fragments (Youle and Karbowski, 2005). These fragments, characterized by reduced membrane potential and increased permeability, release pro-apoptotic factors into the cytoplasm through the cysteine aspartate protease pathway, thereby inducing mitochondrial apoptosis (Youle and Karbowski, 2005). In PC, enhanced mitochondrial fission leads to mitochondrial fragmentation, which is associated with increased tumor cell proliferation, invasion, and resistance to apoptosis. For instance, key regulators of mitochondrial fission such as MTFP1 and MTFP2 are significantly upregulated in PC tissues and correlate with poor patient prognosis (Huangfu et al., 2025). Furthermore, enhanced fission is often accompanied by upregulation of glycolysis, supplying biosynthetic precursors for rapid proliferation. Studies have shown that Drp-1-mediated fission promotes the expression of glycolytic genes in prostate cancer cells, and that inhibition of fission can reverse this process (Baumgartner et al., 2024). Additionally, mitochondrial fragmentation has been implicated in maintaining the self-renewal and tumorigenic potential of cancer stem cell (CSC) subpopulations, playing a critical role in maintaining PC stem cell characteristics (Civenni et al., 2019).

Therapeutic resistance represents a major challenge in PC management, particularly as CRPC frequently develops resistance to conventional therapies such as ADT and PARP inhibitors. Enhanced mitochondrial fission interacts with the AR signaling pathway to promote CRPC progression. In both androgen-sensitive and castration-resistant AR-driven prostate cancers, AR signaling upregulates the expression of Drp-1, which supports cancer cell survival under various metabolic stresses—such as hypoxia and oxidative stress—thereby enhancing cell survival and proliferation. Conversely, inhibition of mitochondrial fission can restore cellular sensitivity to AR inhibitors (Baumgartner et al., 2024; Berger et al., 2025; Lee et al., 2020). For example, in mouse models of CRPC, exercise has been shown to attenuate tumor growth by downregulating the expression of Drp-1 and FIS1, suggesting that the modulation of mitochondrial dynamics can ameliorate treatment response (Berger et al., 2025; Berger et al., 2024). This suggests that the mechanisms of action of some AR-targeting PC therapeutics may also be related to mitochondrial fission.

Mitophagy is the selective removal of damaged mitochondria and is a crucial mechanism for controlling mitochondrial quantity and quality (Onishi et al., 2021). The role of mitophagy in PC is complex. Elevated levels of mitophagy inhibitor LRPPRC are positively correlated with poor prognosis and shorter survival in PC patients, suggesting an anti-cancer effect of mitophagy (Zou et al., 2014). However, mitophagy is a key mechanism for PC cells to resist oxidative stress, implying a pro-cancer role of mitophagy (Balvan et al., 2015). Furthermore, targeting mitophagy influences the efficacy of PARP inhibitors such as Olaparib. Resistance to Olaparib is associated with enhanced mitochondrial function and PINK1 overexpression. As a key regulator of mitophagy, PINK1 upregulation promotes mitochondrial clearance and metabolic adaptation, contributing to drug resistance. Conversely, inhibition of either PINK1 or mitochondrial fission can restore sensitivity to Olaparib (Schaaf et al., 2024). Similarly, DGAT1 inhibition enhances the pro-apoptotic effects of Olaparib by disrupting lipid droplet formation and inducing lipotoxicity—a process further associated with mitochondrial fission and oxidative stress (Morshed et al., 2025). As with the complex multifaceted role of autophagy in tumors, selective autophagy such as mitophagy also appears contradictory in its role in PC, which may be related to specific preclinical models.

Furthermore, there are significant differences in the lipid composition of mitochondrial membranes between normal cells and cancer cells in PC (Zichri et al., 2021). Specifically, increased cardiolipin concentration and decreased phosphatidylcholine levels have been observed in cancer cell mitochondria (Zichri et al., 2021). Abnormalities in mitochondrial membrane composition may contribute to the unique characteristics of mitochondrial dynamics in PC. In conclusion, increasing evidence supports the involvement of mitochondrial dynamics in the development of PC, but further research is needed to better understand their roles in prostate tumorigenesis and progression.

### 6.2 Targeting strategies

**FIGURE 3:**
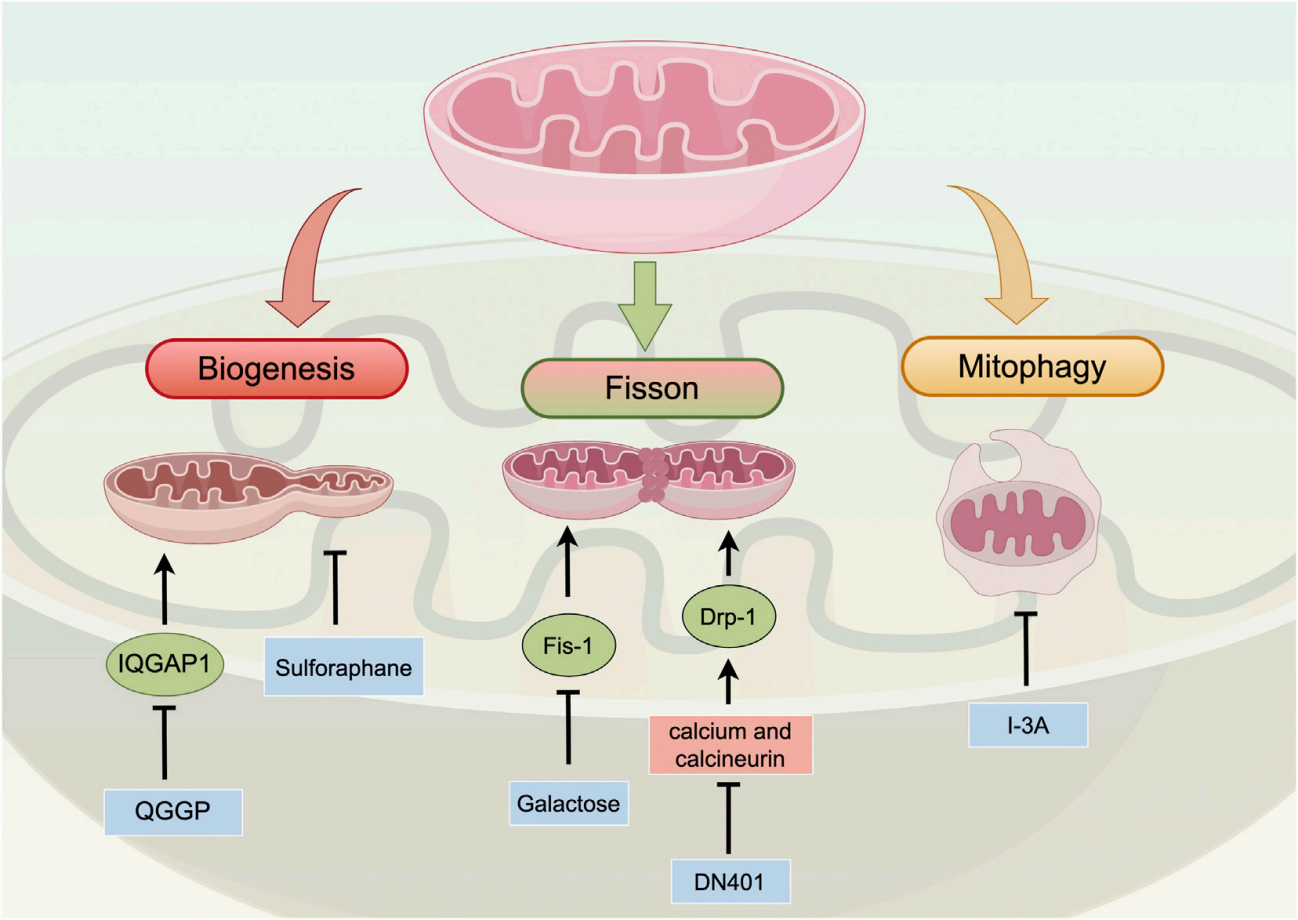
Overview of mitochondrial dynamics in PC. Mitochondrial dynamics, including biogenesis, fusion/fission, and mitophagy, play a crucial role in PC progression. Abnormalities in these processes can promote cancer cell apoptosis and metabolic reprogramming. Mitochondrial biogenesis generates new mitochondria from existing organelles and is associated with malignant tumor phenotypes. Fusion/fission changes mitochondrial morphology and is linked to PC progression and chemotherapy sensitivity. Furthermore, excessive fission triggers mitochondria-mediated cell apoptosis and maintains PC stem cell characteristics. Mitophagy selectively removes damaged mitochondria and has complex roles in PC. Targeting strategies for PC intervention involve natural compounds and inhibitors that modulate mitochondrial dynamics. Sulforaphane and QGGP disrupt mitochondrial biogenesis. DN401 and Galactose induce changes in mitochondrial fission dynamics. I-3A induces immunogenic cell death through mitophagy. Abbreviations: IQGAP1: IQ motif-containing GTPase-activating protein 1 QGGP: Quercetin 3-O-(6″-galactopyranosyl)-β-D-galactopyranoside; Drp-1: dynamin-related protein 1; Fis-1: mitochondrial fission 1 protein; I-3A: Ingenol-3-angelate; DN401: a pan-Hsp90 inhibitor.

Therapeutic strategies targeting mitochondrial dynamics have garnered increasing attention. Various natural compounds and synthetic drugs exert antitumor effects by modulating these dynamic processes (Figure 3; Table 5).

**TABLE 5:** Targeting Mitochondrial dynamics in PC.

Targeted drug or treatment	Primary mechanism	Current limitations/Challenges	Translational readiness	Ref.
QGGP	Inhibits mitochondrial generation by suppressing IQGAP1 and downregulating PGC-1α signaling	Primarily targets CAF-driven biogenesis; direct effect on tumor cell-autonomous process needs study	Preclinical research	Xiong et al. (2025)
Sulforaphane	Disrupts mitochondrial biogenesis processes	Dietary compound; achieving effective anticancer doses may be challenging; mechanism of action is complex	Preclinical research; studied more as a chemopreventive agent	Negrette-Guzman et al. (2017)
Galactose	Affects mitochondrial morphology and Fis-1 protein expression, promoting apoptosis	Unclear specific molecular target; limited efficacy as monotherapy	Preclinical research	Deng et al. (2022)
Hsp90 inhibitors (DN401)	Activates Drp1 via calcium signaling, inducing excessive fission and cell death	Hsp90 is functionally important in normal cells; potential for significant side effects	Preclinical research	Park et al. (2020)
I3A	Triggers mitophagy and immunogenic cell death	Mechanism complex; mitophagy context-dependent (pro-survival or pro-death); requires precise modulation	Preclinical research	Yang et al. (2022), Wang et al. (2022)

Abbreviations: PC: prostate cancer; QGGP: Quercetin 3-O-(6″-galactopyranosyl)-β-D-galactopyranoside; I3A: Ingenol-3-angelate.

Sulforaphane, an isothiocyanate present in many cruciferous vegetables, can induce apoptosis in PC cells by disrupting mitochondrial biogenesis (Negrette-Guzman et al., 2017). Furthermore, QGGP can inhibit mitochondrial biogenesis by targeting IQGAP1 (Xiong et al., 2025).

Galactose inhibits PC progression both *in vitro* and *in vivo*, and its anti-cancer effects are centered around affecting mitochondrial morphology and fission protein expression (Deng et al., 2022). The accumulation of Galactose in PC cells leads to changes in mitochondrial morphology and affects the expression of mitochondrial division protein Fis-1, thereby promoting apoptosis (Deng et al., 2022). Heat shock protein 90 (Hsp90), ATP-dependent molecular chaperones, manage the stability and function of specific proteins that promote growth, survival, and stress adaptation in cancer cells (Taipale et al., 2010). Given the recognition of Hsp90 as a potential target for cancer treatment, numerous inhibitors aimed at cytoplasmic Hsp90 have been formulated as anticancer medications (Xiao and Liu, 2020). Analysis of cancer genome atlas databases shows upregulation of all Hsp90 paralogs in PC (Park et al., 2020). Park et al. found that pan-Hsp90 inhibitor DN401 induces mitochondrial dysfunction in PC, increases cytoplasmic calcium, and activates calcium-dependent phosphatases (Park et al., 2020). Cytoplasmic calcium regulates a range of cellular activities, among which are mitochondrial fusion/fission dynamics that have a close association with the regulation of cell death pathways (Bravo-Sagua et al., 2017). Calcineurin triggers the dephosphorylation of Drp-1), leading to the subsequent relocation of Drp1 to mitochondria and the consequent division of mitochondria (Youle and Karbowski, 2005). Therefore, DN401-induced cytotoxicity depends on cytoplasmic calcium and activation of calcium-dependent phosphatase-mediated mitochondrial fission (Park et al., 2020).

PC patients often do not benefit significantly from immunotherapy, but recent studies suggest that targeting mitochondrial autophagy may change this situation (Klein et al., 2020). Ingenol-3-angelate (I3A) is an emerging anti-tumor drug with dual chemo-immunomodulatory effects, inducing primary necrosis and transient local inflammation in tumor cells (Le et al., 2009). I3A induces immunogenic cell death of PC cells by triggering mitochondrial autophagy and apoptosis, promoting tumor vascular normalization and facilitating extensive infiltration of immune cells into the tumor (Yang et al., 2022). Based on the anti-tumor effects of I3A, Wang et al. developed a dual-targeted delivery system of I3A and doxorubicin, which targets prostate-specific membrane antigen (PSMA) and mitochondria, inhibiting PC growth and eliciting potent anti-tumor immune responses (Wang et al., 2022).

### 6.3 Mitochondrial dynamics: a context-dependent therapeutic target

The roles of mitochondrial dynamics—fusion, fission, and mitophagy—in prostate cancer are supported by seemingly contradictory evidence, reflecting their high context-dependent functionality. For instance, mitophagy can act as a tumor-suppressive mechanism (as suggested by the association between its inhibitor LRPPRC and poor prognosis), yet it also serves as a survival mechanism for cancer cells under stress(Balvan et al., 2015; Zou et al., 2014; Schaaf et al., 2024; Morshed et al., 2025). Similarly, mitochondrial fission promotes proliferation and stem-like properties, whereas fusion has been linked to chemosensitivity in certain settings(Jin et al., 2022; Baumgartner et al., 2024; Berger et al., 2025; Lee et al., 2020).

An important emerging pattern is the close interaction between mitochondrial dynamics and key oncogenic signaling pathways. AR signaling promotes fission through regulation of Drp1, thereby linking metabolic reprogramming to disease progression (Baumgartner et al., 2024; Berger et al., 2025; Lee et al., 2020). This suggests that targeting dynamics could enhance the efficacy of existing therapies, such as AR signaling inhibitors or PARP inhibitors. For example, inhibiting fission or mitophagy has been shown to reverse resistance to Olaparib (Morshed et al., 2025).

A major challenge in therapeutic targeting lies in the precise modulation of these processes. Excessive inhibition of either fission or fusion may exert toxicity in normal cells. Future research must more clearly define whether specific dynamic processes should be activated or suppressed in particular PC subtypes, stages, or treatment contexts. Moreover, developing small-molecule inhibitors that specifically target mitochondrial dynamics proteins and exploring their synergy with immunotherapy represent an exciting new frontier.

## 7 Clinical translation and future directions

The multifaceted role of mitochondria in prostate cancer—spanning metabolic reprogramming, mtDNA mutations, ROS signaling, apoptosis regulation, and mitochondrial dynamics—has been well-established in preclinical studies. However, translating these findings into clinically viable therapies remains challenging. This section outlines the key hurdles in drug development, proposes rational combination strategies, emphasizes biomarker-driven patient stratification, and highlights novel mechanisms and delivery systems that may accelerate the clinical adoption of mitochondrial-targeted therapies.

A primary obstacle in developing mitochondrial-targeted therapies lies in achieving adequate bioavailability and tumor specificity. Many natural compounds and early-generation inhibitors exhibit unfavorable pharmacokinetic profiles, though recent advances are promising. For instance, the novel mitochondrial RNA polymerase inhibitor YH-0623 has demonstrated high oral bioavailability and significant antitumor efficacy in preclinical models, representing a step forward in targeting mitochondrial transcription (Li et al., 2025). To enhance specificity, nanocarriers functionalized with prostate-specific membrane antigen (PSMA)-targeting ligands offer a viable strategy for selective drug delivery to prostate cancer cells (Li et al., 2025). Systemic energy toxicity also poses a major concern, as tissues such as the heart and brain rely heavily on OXPHOS (Liu et al., 2025; Wang et al., 2025). This underscores the necessity of tumor-selective targeting, which may be achieved by exploiting the metabolic heterogeneity of prostate cancer. The clinical-grade OXPHOS inhibitor IACS-10759, for example, shows efficacy in OXPHOS-dependent models but not in glycolytic subtypes, suggesting that metabolic subtyping could help mitigate systemic toxicity (Mossa et al., 2023).

The metabolic plasticity of cancer cells frequently leads to resistance against single-agent therapies, making rational combination approaches essential. Co-targeting OXPHOS and glycolysis represents a logical strategy to prevent compensatory metabolic shifts. The natural compound juglone suppresses both pathways, while the combination of 2-deoxy-D-glucose and buforin IIb synergistically induces apoptosis in prostate cancer models (Hu et al., 2023; Jang et al., 2015). Mitochondrial-targeting agents can also resensitize tumors to conventional therapies. PARP inhibitors like rucaparib induce mitochondrial fragmentation via Ca^2+^/CaMKII/Drp1 signaling, suggesting potential synergy with mitochondrial destabilizers (Arbini et al., 2013). Additionally, sequential treatment regimens—such as first inducing OXPHOS dependence before administering OXPHOS inhibitors—may maximize therapeutic efficacy through carefully timed interventions.

Given the context-dependent efficacy of mitochondrial-targeted therapies, robust biomarkers are indispensable for patient selection. Beyond traditional parameters like Gleason score and AR status, molecular markers such as PGC-1α and ERRα expression may identify tumors reliant on mitochondrial metabolism (Varuzhanyan et al., 2024; Ma et al., 2025). Liquid biopsy approaches offer dynamic monitoring capabilities, with circulating cell-free mtDNA levels and specific mutations correlating with tumor burden and progression. The detection of SDHB mRNA in extracellular vesicles shows particular promise for early identification of resistance to AR signaling inhibitors (Kato et al., 2024).

Future progress will depend on innovations across multiple fronts. Advanced delivery systems, particularly smart nanocarriers capable of dual targeting to both tumor-specific antigens and mitochondria, can enhance therapeutic precision while minimizing off-target effects. Well-designed, biomarker-enriched clinical trials are essential for validating these approaches, with adaptive platform trials offering efficient models for evaluating multiple therapeutic strategies simultaneously. The exploration of novel biological mechanisms continues to reveal unexpected therapeutic opportunities. Recent findings demonstrate that prostate cancer cells can acquire functional mitochondria from neurons via tunneling nanotubes, promoting metastatic progression—a process that could be therapeutically targeted (Hoover et al., 2025). Furthermore, mtDNA release from senescent cells enhances immunosuppression through the cGAS-STING pathway in myeloid-derived suppressor cells, suggesting that mitochondrial stress inducers may synergize with immunotherapy by remodeling the tumor immune landscape (Lai et al., 2025).

In conclusion, mitochondrial targeting holds significant potential for advancing prostate cancer treatment. Realizing this potential will require a multidisciplinary strategy that integrates innovative drug design, rational combination therapies, validated biomarkers, and the exploration of emerging biological mechanisms. By systematically addressing these priorities, mitochondrial biology can transition from a compelling research paradigm to a cornerstone of precision oncology in prostate cancer, ultimately delivering more effective and personalized treatments to patients.

## 8 Conclusion

The role of mitochondria in PC has surfaced as a compelling research domain with significant implications for diagnosis and treatment. The multifarious involvement of mitochondria in energy metabolism, mtDNA regulation, oxidative stress, apoptosis, and mitochondrial dynamics underscores their critical influence on PC biology.

Mitochondrial-targeted interventions hold substantial potential for the development of innovative therapeutic strategies. Disrupting metabolic reprogramming and selectively inhibiting OXPHOS may offer a means to specifically target PC cells while sparing normal tissues. Moreover, understanding the impact of mtDNA on mitochondrial function can lead to personalized treatment approaches tailored to individual patients’ needs. Restoring redox balance and selectively inducing apoptosis in PC cells through targeted modulation of ROS levels could open new opportunities for treatment. Furthermore, deciphering the mechanisms underlying mitochondrial-mediated apoptosis, such as dysregulation of MOMP and alterations in Bcl-2 family proteins, may pave the way for the development of strategies to enhance treatment outcomes. The intricate interplay between mitochondrial dynamics and PC progression provides another promising area for exploration. Targeting mitochondrial fusion, fission, and mitophagy processes may offer novel therapeutic strategies to induce apoptosis and inhibit tumor growth. By manipulating these dynamic processes, it may be possible to disrupt the survival mechanisms of PC cells and enhance treatment efficacy.

However, further research is required to fully comprehend the complex molecular mechanisms underpinning mitochondrial involvement in PC. Preclinical models that accurately mimic the disease complexity, along with clinical trials evaluating the safety and efficacy of mitochondrial-targeted therapies, are essential steps towards translating these findings into clinical practice. In conclusion, the burgeoning body of evidence underscoring the pivotal role of mitochondria in PC unveils exciting possibilities for the development of targeted therapeutic approaches. Continued research in this field will contribute to a deeper understanding of PC biology and pave the way for more effective treatments, ultimately improving patient outcomes.
